# Supplementary material for: The Discourse Profile in Corticobasal Syndrome: A Comprehensive Clinical and Biomarker Approach
Source: Brain Sci. 2022 Dec 12;12(12):1705. doi: 10.3390/brainsci12121705 (PMC9775929; doi:10.3390/brainsci12121705)
Supplement: Supplementary file 1 [file brainsci-12-01705-s001.zip › brainsci-2058356-supplementary.pdf]

Table S1. Individual performance on discursive measures related to "Speech rate and speech sound errors" and "Other disruptions to fluency"

	Speech rate and speech sound errors			Other disruption to fluency				
Subject	Total number of words	Speech production rate	Phonological paraphasias phw	Distortions phw	False starts phw	Repaired sequences phw	Filled pauses phw	Incomplete sentences phw
**1**	59	1.26	0.00	1.69	3.39	13.56	1.69	5.08
**2**	73	1.20	0.00	0.00	4.11	2.74	4.11	0.00
**3**	62	0.61	0.00	0.00	3.23	17.74	4.84	9.68
4	68	1.21	0.00	0.00	1.47	20.59	4.41	1.47
5	26	0.90	0.00	0.00	0.00	0.00	0.00	0.00
6	28	0.70	0.00	3.57	0.00	14.29	0.00	21.43
7	98	1.20	0.00	3.06	1.02	4.08	0.00	0.00
**8**	68	0.73	0.00	2.94	1.47	4.41	1.47	1.47
9	71	1.00	0.00	15.49	11.27	4.23	4.23	0.00
10	28	0.49	0.00	32.14	3.57	0.00	0.00	10.71
11	24	0.60	0.00	0.00	0.00	8.33	4.17	4.17
12	61	0.97	0.00	0.00	0.00	9.84	0.00	0.00
13	34	0.62	0.00	0.00	0.00	2.94	0.00	0.00
14	47	0.64	0.00	0.00	0.00	10.64	0.00	2.13
mean (SD)	53.36 (22.52)	0.87 (0.27)	0.0 (0.0)	4.21 (9.03)	2.11 (3.06)	8.1 (6.55)	1.78 (2.07)	4.01 (6.17)
min-max	24-98	0.49-1.26	0-0	0.0-32.14	0.0-11.27	0.0-20.59	0.0-4.84	0.0-21.43

Note: Subjects identified with bold faced numbers pertain to CBS-AD subgroup. Abbreviations: phw = per hundred words.

Table S2. Individual performance on discursive measures related to "Lexical-semantic level"

Subject	Open class proportion	Verb proportion	Lexical-semantic errors phw	% CIU
**1**	0.37	0.52	3.39	68.25
**2**	0.74	0.44	2.74	94.52
**3**	0.63	0.45	17.74	45.00
4	0.68	0.39	2.94	77.22
5	0.73	0.36	0.00	64.71
6	1.00	0.27	3.57	71.43
7	0.85	0.45	2.04	91.84
**8**	0.62	0.44	1.47	67.90
9	0.54	0.64	0.00	80.52
10	0.75	0.33	0.00	75.00
11	0.85	0.60	12.50	73.08
12	0.65	0.34	1.64	84.48
13	0.48	0.38	14.71	31.58
14	0.62	0.43	0.00	88.24
mean (SD)	0.68 (0.16)	0.43 (0.10)	4.48 (5.92)	72.41 (17.26)
min-max	0.37-1.0	0.27-0.64	0.0-17.74	31.58-94.52

Note: Subjects identified with bold faced numbers pertain to CBS-AD subgroup. Abbreviations: phw = per hundred words; CIU = correct information unit.

Table S3. Individual performance on discursive measures related to "Syntactic structure and complexity"

Subject	Number of utterances	Number of sentences	Mean length of sentences	Proportion of sentences	Number of embeddings phw	Syntax production rate	Morphosyntactic errors rate
**1**	12.00	9.00	5.78	0.75	1.69	0.88	0.02
**2**	9.00	9.00	8.11	1.00	4.11	1.00	0.00
**3**	14.00	8.00	4.50	0.57	1.61	0.58	0.00
4	8.00	7.00	9.00	0.88	1.47	0.93	0.02
5	6.00	5.00	4.80	0.83	0.00	0.92	0.04
6	10.00	4.00	4.75	0.40	0.00	0.68	0.00
7	21.00	20.00	4.80	0.95	0.00	0.98	0.00
**8**	16.00	15.00	4.33	0.94	1.47	0.96	0.00
9	16.00	15.00	4.47	0.94	0.00	0.94	0.01
10	9.00	2.00	5.00	0.22	0.00	0.36	0.00
11	7.00	4.00	5.25	0.57	0.00	0.88	0.00
12	11.00	10.00	5.60	0.91	0.00	0.92	0.00
13	8.00	5.00	5.60	0.63	0.00	0.82	0.00
14	8.00	7.00	6.14	0.88	0.00	0.91	0.07
mean (SD)	11.07 (4.27)	8.57 (5.05)	5.58 (1.38)	0.75 (0.23)	0.74 (1.21)	0.84 (0.18)	0.01 (0.02)
min-max	6-21	2-20	4.3-9.0	0.2-1.0	0.0-4.11	0.36-1.0	0-0.07

Note: Subjects identified with bold faced numbers pertain to CBS-AD subgroup. Abbreviations: phw = per hundred words.

Table S4. Demographic and clinical characteristics of controls, CBS-non-AD and CBS-AD patients

Mean(SD)	**CBS-non-AD (n=10)**	**CBS-AD (n=4)**	**CG (n=15)**	**Statistic****	**p-value**
Age (y)	66.4 (8.3)	71.5 (8.7)	67.3 (8.1)	0.585	0.746
Education (y)	9.3 (5.9)	7.5 (6.0)	9.3 (5.4)	0.266	0.875
Sex (F/M)	5 / 5	4 / 0	10 / 5	3.180	0.203
Hand dominance (R/L)	8 / 2	4 / 0	14 / 1	1.686	0.430
Symptoms' duration (y)	4.7 (2.4)	4.5 (1.2)	--	-	0.770*
CDR	1.9 (0.7)	2.0 (0.8)	--	-	0.941*
FAQ	18.6 (7.6)	23.0 (8.4)	--	-	0.337*
NPI	12.9 (8.7)	26.7 (27.1)	--	-	0.394*
H&Y	3.0 (1.3)	2.7 (1.7)	--	-	0.774*

Note: Data reported as mean (SD) with minimum and maximum values below; Comparison's analysis across all groups were performed using Kruskal-Wallis. * CBS-non-AD vs CBS-AD comparison using Mann-Whitney test. Sex and Hand dominance were compared across groups using Chi-squared test. ** This column presents the chi-squared statistics from Kruskal-Wallis test and the values from the Chi-square test. Abbreviations: CBS = corticobasal syndrome, CBS-non-AD = corticobasal syndrome not related to Alzheimer's disease, CBS-AD = corticobasal syndrome related to Alzheimer's disease; SD = standard deviation, y = years, CDR = Clinical Dementia Rating, FAQ = Functional activities questionnaire, NPI = Neuropsychiatric Inventory, H&Y = Hoehn &Yahr scale.

Table S5. Performance on cognitive and language tests across diagnostic groups

Mean (SD)	**CBS-non-AD (n=10)**	**CBS-AD (n=4)**	**CG (n=15)**	**Statistic****	**p-value**
**ACE-R**					
Total score	48.5 (18.4)	32.8 (19.5)	86.1 (9.0)	18.833	**<0.001**[ab]
Attention	12.3 (3.0)	7.0 (3.7)	16.9 (1.3)	16.530	**<0.001**[ab]
Memory	11.6 (6.7)	5.0 (5.3)	20.3 (5.3)	14.138	**0.001**[ab]
Fluency	2.7 (2.7)	2.3 (2.6)	10.3 (2.2)	20.225	**<0.001**[ab]
Language	15.4 (6.4)	15.0 (8.0)	24.4 (2.4)	11.889	**0.003**[a]
Visuospatial	6.5 (4.0)	3.5 (0.6)	14.3 (1.7)	20.890	**<0.001**[ab]
**WAB-R**					
Aphasia Quotient	83.8 (11.5)	81.0 (13.6)	98.2 (1.6)	17.406	**<0.001**[ab]
Spont speech	16.9 (2.3)	16.5 (2.9)	19.9 (0.3)	18.059	**<0.001**[ab]
Aud comp	8.6 (1.6)	8.7 (1.2)	9.9 (0.1)	9.040	**0.011**[b]
Repetition	8.8 (0.9)	8.5 (1.3)	9.7 (0.3)	12.107	**0.002**[ab]
Naming	7.3 (2.5)	6.9 (1.8)	9.6 (0.6)	13.527	**0.001**[ab]

Note: Data reported as mean (SD) with minimum and maximum values below; Comparison's analysis across all groups were performed using Kruskal-Wallis. * CBS-non-AD vs CBS-AD comparison using Mann-Whitney test. Sex and Hand dominance were compared across groups using Chi-squared test. ** This column presents the chi-squared statistics from Kruskal-Wallis test and the values from the Chi-square test.; a = CBS-non-AD vs. CG p-value < 0.05, b = CBS-AD vs. CG p-value < 0.05, c = CBS-non-AD vs. CBS-AD p-value < 0.05. Bold faced values are statistically significant according to P values. Abbreviations: CBS = corticobasal syndrome, CBS-non-AD = corticobasal syndrome not related to Alzheimer's disease, CBS-AD = corticobasal syndrome related to Alzheimer's disease; ACE-R = Addenbrooke's Cognitive Examination-revised; WAB-R = Western Aphasia Battery – Revised; SD = standard deviation, Spont speech = Spontaneous speech, Aud comp = Auditory comprehension.

Table S6. Performance on discursive measures related to "Speech rate and speech sound errors" and "Other disruptions to fluency" by diagnostic group

Mean (SD)<br>Min-Max	**CBS-non-AD (n=10)**	**CBS-AD (n=4)**	**CG (n=15)**	**Statistic****	**p-value**
**Speech rate and speech sound errors**					
Number of words	48.5 (25.1)<br>24-98	65.5 (6.2)<br>59-73	72.9 (27.3)<br>36-131	5.468	0.065
Speech prod rate	0.8 (0.2)<br>0.5-1.2	0.9 (0.3)<br>0.6-1.3	1.4 (0.3)<br>0.8-1.8	15.646	**<0.001**[a]
Phon paraphasias phw	0.0 (0.0)<br>0-0	0.0 (0.0)<br>0-0	0.0 (0.0)<br>0-0	<0.001	1.000
Distortions phw	5.4 (10.5)<br>0.0-32.1	1.2 (1.4)<br>0.0-2.9	0.0 (0.3)<br>0.0-1.4	5.680	0.058
**Other disruption to fluency**					
False starts phw	1.73 (3.5)<br>0.0-11.3	3.0 (1.1)<br>1.4-4.1	1.8 (2.6)<br>0.0-10.0	4.442	0.109
Repaired sequences phw	7.5 (6.6)<br>0.0-20.6	9.6 (7.2)<br>2.7-17.7	5.5 (3.3)<br>0.8-11.8	0.855	0.652
Filled pauses phw	1.3 (2.0)<br>0.0-4.4	3.0 (1.7)<br>1.5-4.8	0.7 (1.4)<br>0.0-4.1	7.444	**0.024**[b]
Incomplete sentences phw	3.4 (6.7)<br>0.0-21.4	4.1 (4.3)<br>0.0-9.7	0.6 (1.2)<br>0.0-4.2	4.238	0.120

Note: Data reported as mean (SD) with minimum and maximum values below; Comparison's analysis across all groups were performed using Kruskal-Wallis. * CBS-non-AD vs CBS-AD comparison using Mann-Whitney test. Sex and Hand dominance were compared across groups using Chi-squared test. ** This column presents the chi-squared statistics from Kruskal-Wallis test and the values from the Chi-square test; a = CBS-non-AD vs. CG p-value > 0.05, b = CBS-AD vs. CG p-value > 0.05, c = CBS-non-AD vs. CBS-AD p-value > 0.05. Bold faced values are statistically significant according to P values. Abbreviations: phw = per hundred words, CBS = corticobasal syndrome patients, CBS-non-AD = corticobasal syndrome not related to Alzheimer's disease patients, CBS-AD = corticobasal syndrome related to Alzheimer's disease patients; SD = standard deviation, s = seconds, Speech prod rate = Speech production rate, Phon paraphasias = Phonological paraphasias.

Table S7. Performance on discursive measures related to "Lexical-semantic level" by diagnostic group

Mean(SD)<br>Min-Max	**CBS-non-AD (n=10)**	**CBS-AD (n=4)**	**CG (n=15)**	**Statistic****	**p-value**
Open-class proportion	0.7 (0.1)<br>0.5-1.0	0.6 (0.1)<br>0.4-0.7	1.0 (0.2)<br>0.6-1.3	12.371	**0.002**[ab]
Verb proportion	0.4 (0.1)<br>0.3-0.6	0.4 (0.0)<br>0.4-0.5	0.4 (0.0)<br>0.3-0.6	1.946	0.378
Lexical-semantic errors phw	3.7 (5.4)<br>0.0-14.7	6.3 (7.6)<br>1.5-17.7	0.7 (1.5)<br>0.0-5.6	8.743	**0.013**[b]
CIU %	73.8 (16.9)<br>31.6-91.8	68.9 (20.2)<br>45.0-94.5	84 (6.9)<br>70.8-92.0	4.579	0.101

Note: Data reported as mean (SD) with minimum and maximum values below; Comparison's analysis across all groups were performed using Kruskal-Wallis. * CBS-non-AD vs CBS-AD comparison using Mann-Whitney test. Sex and Hand dominance were compared across groups using Chi-squared test. ** This column presents the chi-squared statistics from Kruskal-Wallis test and the values from the Chi-square test; a = CBS-non-AD vs. CG p-value > 0.05, b = CBS-AD vs. CG p-value > 0.05, c = CBS-non-AD vs. CBS-AD p-value > 0.05. Bold faced values are statistically significant according to P values. Abbreviations: phw = per hundred words, CIU = correct information units, CBS = corticobasal syndrome patients, CBS-non-AD = corticobasal syndrome not related to Alzheimer's disease patients, CBS-AD = corticobasal syndrome related to Alzheimer's disease patients; SD = standard deviation.

Table S8. Performance on discursive measures related to "Syntactic structure and complexity" by diagnostic group

Mean (SD)<br>Min-Max	**CBS-non-AD (n=10)**	**CBS-AD (n=4)**	**CG (n=15)**	**Statistic****	**p-value**
Number of utterances	10.4 (4.6)<br>6-21	12.8 (3.0)<br>9-16	12.7 (4.2)<br>7-19	2.521	0.284
Number of sentences	7.9 (5.6)<br>2-20	10.2 (3.2)<br>8-15	8.8 (4.4)<br>0-16	1.537	0.464
Embeddings phw	0.1 (0.5)<br>0.0-1.4	2.2 (1.3)<br>1.5-4.1	2.4 (1.8)<br>0.0-4.7	11.531	**0.003**[a]
Mean length of sentences	5.5 (1.3)<br>4.5-9.0	5.7 (1.7)<br>4.3-8.1	6.4 (2.8)<br>3.8-11.3	1.955	0.376
Proportion of sentences	0.7 (0.2)<br>0.2-0.9	0.8 (0.2)<br>0.6-1.0	0.6 (0.2)<br>0.0-1.0	1.567	0.457
Syntax production rate	0.8 (0.1)<br>0.4-1.0	0.8 (0.2)<br>0.6-1.0	0.7 (0.2)<br>0.0-1.0	0.410	0.815
Morphosyntactic errors phw	0.0 (0.0)<br>0-0	0.0 (0.0)<br>0-0	0.0 (0.0)<br>0-0	0.716	0.699

Note: Data reported as mean (SD) with minimum and maximum values below; Comparison's analysis across all groups were performed using Kruskal-Wallis. * CBS-non-AD vs CBS-AD comparison using Mann-Whitney test. Sex and Hand dominance were compared across groups using Chi-squared test. ** This column presents the chi-squared statistics from Kruskal-Wallis test and the values from the Chi-square test; a = CBS-non-AD vs. CG p-value > 0.05, b = CBS-AD vs. CG p-value > 0.05, c = CBS-non-AD vs. CBS-AD p-value > 0.05. Bold faced values are statistically significant according to P values. Abbreviations: phw = per hundred words, CBS = corticobasal syndrome patients, CBS-non-AD = corticobasal syndrome not related to Alzheimer's disease patients, CBS-AD = corticobasal syndrome related to Alzheimer's disease patients, SD = standard deviation.
